# Partial calcanectomy and Ilizarov external fixation may reduce amputation need in severe diabetic calcaneal ulcers

**DOI:** 10.1080/2000625X.2017.1264699

**Published:** 2017-01-20

**Authors:** Mehmet Orçun Akkurt, Ismail Demirkale, Ali Öznur

**Affiliations:** ^a^ Departments of Orthopaedics and Traumatology, Ankara Yenimahalle Education and Research Hospital, Ankara, Turkey; ^b^ Keçiören Education and Research Hospital, Department of Orthopaedics, Ankara, Turkey; ^c^ TOBB University, ETÜ Hospital, Department of Orthopaedics, Ankara, Turkey

**Keywords:** Diabetic foot ulcer, diabetes mellitus, Ilizarov technique, external fixation, surgical debridement

## Abstract

**Objective**: The treatment of diabetic hindfoot ulcers is a challenging problem. In addition to serial surgical debridements, hyperbaric oxygen therapy and local wound care play important roles in the surgeon’s armamentarium, for both superficial infection and gangrene of the soft tissue, often complicated by osteomyelitis of the calcaneus. The purpose of this study was to evaluate the results of an aggressive approach from diagnosis to treatment of calcaneal osteomyelitis in foot-threatening diabetic calcaneal ulcers.

**Methods**: The study included 23 patients with diabetic hindfoot ulcers who were treated with radical excision of the necrotic tissue and application of circular external fixation. The treatment protocol was a combination of magnetic resonance imaging (MRI)-guided debridement of the necrotic tissues and application of an Ilizarov external fixator in plantarflexion to decrease the soft-tissue defect. Primary outcome measures were total cure of infection and obvious healing of the osteomyelitis at 12 weeks determined by MRI, and clinical cure through objective assessment of the appearance of the wound.

**Results**: The wounds healed in 18 of the 23 patients (78%), partial recovery occurred and subsequent flap operation was performed in three patients (13%), and below-the-knee amputation was performed in two patients (9%).

**Conclusions**: This surgical protocol is effective in ameliorating diabetic hindfoot ulcers with concomitant calcaneal osteomyelitis, and satisfactorily reduces the need for amputation.

## Introduction

Diabetes mellitus has been estimated to affect 11 million patients in the USA, of whom approximately 25% will develop foot problems [1]. Furthermore, it has been shown that at least 15% of hospital admissions are directly related to diabetic foot infections [1,2]. Superficial diabetic foot ulcers can be treated with non-operative techniques such as wound dressings, total contact cast, and serial wound debridements [3,4]. The major consequence of a non-healing ulcer of the foot in a diabetic patient is amputation. The incidence of foot ulcers in diabetic patients is as high as 32% and the 5 year rate of survival after amputation is very low; therefore, the diagnosis and management of ulcers and underlying osteomyelitis in the diabetic foot are crucial [5,6].

The radiological examination of a diabetic foot ulcer is of paramount importance. Although positive plain radiographs and the probe-to-bone (PTB) test have been reported to be sufficient to make the diagnosis, preoperative magnetic resonance imaging (MRI) mapping of the wound to determine the severity of the ulcer and the extent of osteomyelitis involvement helps the surgeon in decision making regarding the surgical margin [7,8]. A combination of severe heel pad ulceration with underlying calcaneal osteomyelitis requires radical debridement of unviable tissues, and partial or total calcanectomy with long-term intravenous antibiotic therapy [9,10]. To avoid amputation, adequate closure of the wound after surgical debridement is a necessity.

Only a few studies, with relatively small sample sizes, have evaluated the effectiveness of MRI-guided radical debridement and circular external fixation for wound closure in diabetic calcaneal ulcers. Furthermore, no study has evaluated the effectiveness of MRI-guided surgical debridement and Ilizarov fixation method for pedal ulcers. Therefore, the aim of this study was to evaluate the efficiency of MRI-guided debridement and Ilizarov fixation, and to report the outcomes of this protocol applied to patients with pedal ulcers and concomitant calcaneal osteomyelitis.

## Materials and methods

This observational case series comprised a retrospective review of prospectively collected data of patients with severe heel pad ulcer. All patients were treated by the senior author (AÖ) from 2007 to 2012. All patients had diabetes mellitus, 12 were male and 11 were female, and the average age was 62 years (range 47–82 years). All patients had a history of failed treatments such as local wound care, hospitalization for intravenous antibiotic treatment, and serial debridements of the necrotic tissues.

Approval was sought and obtained from the hospital institutional review board, and the research was performed in accordance with the 1964 Declaration of Helsinki ethical standards. Before surgery, informed consent for study participation was obtained from all patients. The treatment protocol was a combination of MRI-guided debridement of the necrotic tissues and the application of a circular external fixator to decrease the soft-tissue defect. Inclusion criteria were treatment-resistant deep heel pad ulcerations with calcaneal osteomyelitis, and patients who revealed monophasic or biphasic wave patterns on Doppler ultrasonography examination. The exclusion criterion was a fixed ankle deformity of the ulcerated diabetic foot. A total of 23 patients met the criteria.

All patients had severe pedal ulcer with underlying calcaneal osteomyelitis and were using a walker as a supporting ambulation aid. Patients had been recommended amputation at other centers. All patients had various degrees of diminished perception of light touch in a stocking distribution. Owing to end-stage renal disease, four patients (17%) required hemodialysis during their treatment. On first clinical presentation, no patient was receiving any antibiotic therapy. All patients underwent the PTB test, which was applied using a sterile probe to gently explore the wound. Test positivity was defined as palpating calcaneus and all patients had a positive PTB test. Bone specimens were then obtained by curettage or rongeur and specimens were transferred to the clinical microbiology laboratory. Parenteral culture-specific antibiotic therapy was administered under the supervision of the infectious disease specialists according to the results of the culture medium.

The extent of the surgical debridement was based on MRI findings. MRI studies were performed on a 1.5 Tesla system (Intera 1.5 T; Philips Medical Systems, Best, The Netherlands). Multiple sequences were obtained. In all studies, a combination of spin-echo T1 and a water-weighted sequence (T2 fat-saturated and/or short tau inversion recovery) was used in at least two planes. Although T2-weighted images are considered the most sensitive, T1-weighted sections that are more indicative of osteomyelitis were used because the loss of the fatty marrow signal represents true infiltration of the bone by the infectious process.

**Figure 1.**
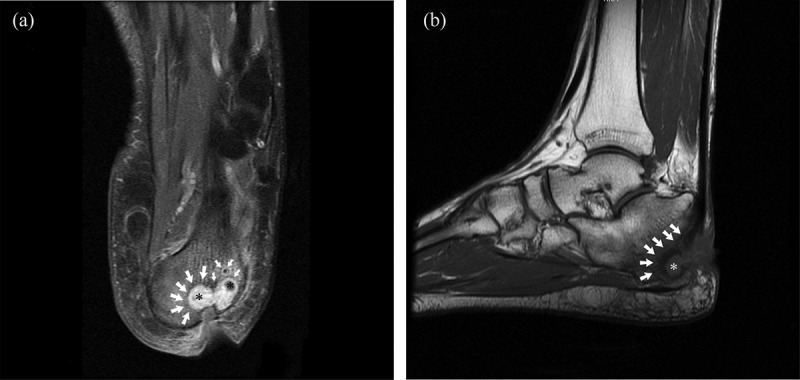
The planned resection level (white arrows) of the osteomyelitis on sagittal (a) and coronal (b) magnetic resonance imaging sections covering the abscess (asterisks). Actual resection can be predicted by including a few millimeters of bone covering the hypointense area over the nidus.

**Figure 2.**
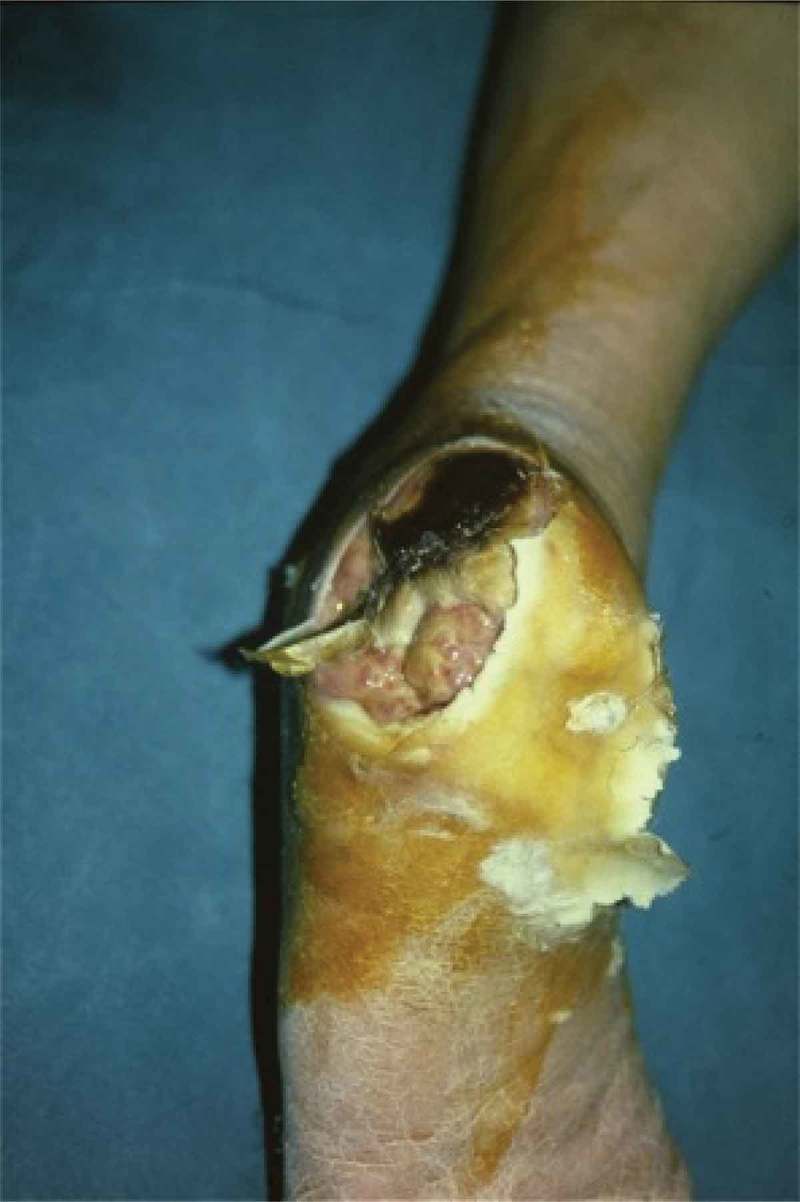
A 72-year-old male presented with a large diabetic foot ulcer over the left heel.

**Figure 3.**
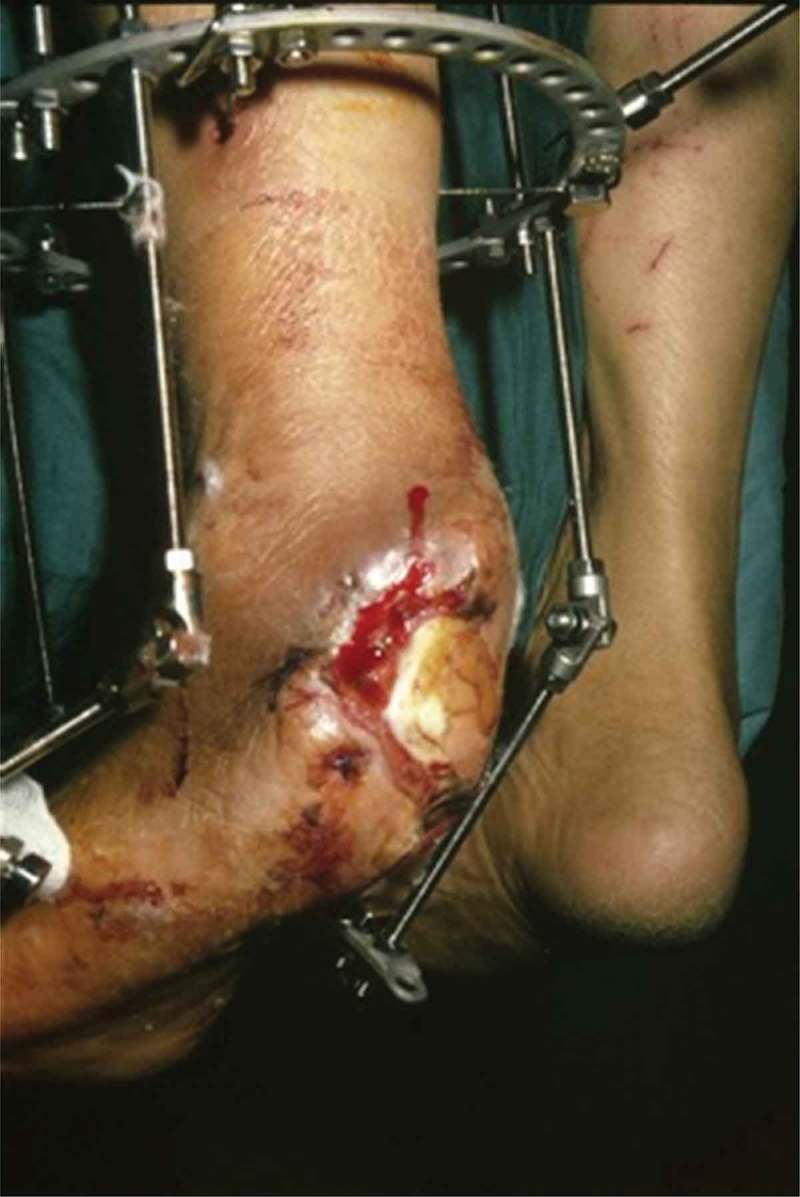
The culture was positive for *Staphylococcus aureus* and *Pseudomonas aeruginosa*. After surgical debridement and partial calcanectomy, an Ilizarov external fixator with hinges was applied to decrease the soft-tissue defect in the equinus position.

**Figure 4.**
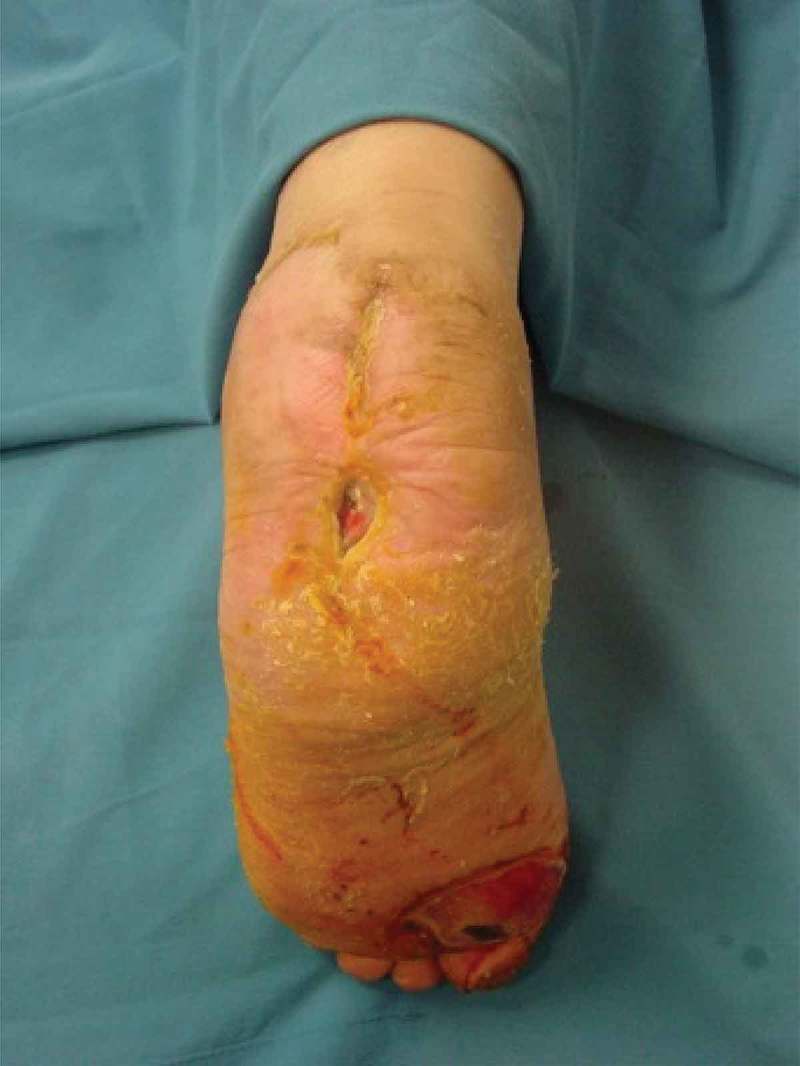
Plantarflexion was gradually decreased and the external fixator was removed in the postoperative 8th week. Clinical outcome with an uneventful recovery.

The procedures were performed under either a regional popliteal block or ankle block anesthesia. The patients were placed supine on a radiolucent operating table to allow access for an image intensifier. No tourniquet was required during the surgical procedure. First, the granulation tissue at the base of the ulcer that was seen as a hyperintense area on T2-weighted images with intense peripheral enhancement on T1-weighted images was debrided. Then, the calcaneal resection was deepened up to the level where the hypointensity on T1-weighted images and hyperintensity on fluid-sensitive images were not visualized (Figure 1(a,b)). Primary closure over the suction drain was applied to 14 wounds. Partial closure to allow for continued drainage and serial surgical debridements was applied to nine wounds. All patients underwent Ilizarov external fixator application with hinges in the equinus position to decrease the soft-tissue defect (Figure 2). Plantarflexion was decreased gradually by 1° per day during the healing period beginning from the postoperative second week and was stopped when a neutral ankle was obtained (Figures 3 and 4). The pin site infections were defined as minor or major according to the Checketts–Otterburn classification [11]. The external fixator was removed after a mean of 8.2 weeks (range 7–10 weeks) and a custom-made ankle–foot orthosis with a soft inner foam lining and a dense outer foam heel was ordered.

Patients were followed up at intervals of three times a week, with wound and pin site care applied during visits. Primary outcome measures were eradication of the infection and obvious healing of the osteomyelitis with MRI findings in the 12th week and clinical cure according to the Wagner classification [12]. Preoperative MRI and postoperative control MRI at the 12th week were examined to delineate the extent of osteomyelitis and abscess localization and to evaluate the effect of treatment on calcaneal osteomyelitis. The mean follow-up duration was 18.2 weeks (range 12–23 weeks) for this study.

## Results

The outcome data are presented in Table 1. Wagner grade II pedal ulcer was determined in 11 patients and Wagner type III in 12 patients. Of these, osteomyelitis was diagnosed in 17 patients. Preoperative evaluation of the extent of the infection almost totally guided the resection procedure of the bone in all patients. The mean size of calcaneal osteomyelitis was 8.73 cm^3^ (range 3–18 cm^3^). Although the exact size of the infection was adequately determined preoperatively, the size of resection exceeded the previously planned resection by up to 0.5 cm in depth to prevent blurring in the separation of healthy from infected tissue in all cases with osteomyelitis. Consequently, actual resection was deepened until fibrosing spongy osseous tissue was totally excised and viable osseous tissue was seen. This enabled radical resection of the infection to be made. Thus, preoperative MRI-guided resection plus a maximum 0.5 cm of resection in depth as far as healthy osseous tissue was sufficient in all patients. Owing to recalcitrant infection by resistant *Pseudomonas aeruginosa* pathogens despite the MRI-guided radical debridement, below-the-knee transtibial amputation was applied to two patients.

**Table 1.** The outcome data of the patients.

Participant	Organism	Ulcer size (cm)	COM	WG	OM	BR	Clinical cure	SI
1	*K. pneumoniae*	6 × 6.5	3 × 3.5 × 1.5	III	+	3 × 4	Partial	Flap
2	*S. aureus*	7 × 6.5	2 × 2.5 × 1	II	+	2 × 3	Complete	
3	*Ps. aeruginosa*	5 × 6.5		II	−		Complete	
4	*S. aureus*	5 × 7.0	3 × 2.5 × 1.7	III	+	3 × 3	Complete	
5	*S. aureus/Ps. aeruginosa*	6 × 6.5	2.5 × 2.5 × 1.5	II	+	3 × 3	No	BKA
6	*S. aureus*	5 × 6.5		II	−		Complete	
7	*K. pneumoniae*	6 × 7.0	3 × 2 × 1.5	III	+	3 × 2	Complete	
8	*E. coli*	6 × 6.5		II	−		Partial	Flap
9	*S. aureus*	8 × 9.0	2 × 2 × 1	II	+	2 × 2	Complete	
10	*Ps. aeruginosa*	6 × 6.5		II	−		Complete	
11	*S. aureus*	9 × 11	2.5 × 3 × 2	III	+	3 × 3	Complete	
12	*Ps. aeruginosa*	7 × 10	2.5 × 2 × 1.1	III	+	3 × 2.5	Partial	Flap
13	*S. aureus*	7 × 9.5	2 × 2 × 1.2	III	+	2.5 × 2.5	Complete	
14	*Ps. aeruginosa*	6 × 7.5	3 × 2.5 × 0.5	III	+	3 × 3	Complete	
15	*K. pneumoniae*	8 × 8.5	3 × 3 × 1.8	III	+	3 × 3.5	Complete	
16	*S. aureus/Ps. aeruginosa*	7 × 6.5		II	−		Complete	
17	*S. aureus/Ps. aeruginosa*	7 × 8.0	2.5 × 2.5 × 1	III	+	3 × 3	No	Flap
18	*S. aureus*	8 × 6.5	3.5 × 2 × 1.2	II	+	4 × 2	Complete	
19	*S. aureus*	6 × 6.5		II	−		Complete	
20	*Ps. aeruginosa*	8 × 9.5	1.5 × 2.5 × 0.8	III	+	2 × 3	Complete	
21	*S. aureus/Ps. aeruginosa*	8 × 10	2 × 2.5 × 1	III	+	3 × 2.5	Complete	
22	*S. aureus/Ps. aeruginosa*	7 × 6.5	3 × 3 × 2	II	+	3 × 3	Complete	
23	*Ps. aeruginosa*	7 × 7.5	2.5 × 3 × 1.6	III	+	3 × 3	Complete	

COM = calcaneal osteomyelitis; WG = Wagner grade; OM = Osteomyelitis; BR = bone resection; SI = secondary intervention; *K. pneumoniae* = *Klebsiella pneumoniae; S. aureus* = *Staphylococcus aureus; Ps. aeruginosa* = *Pseudomonas aeruginosa; E. coli* = *Escherichia coli*; BKA = below-knee amputation.

The size of the calcaneal ulcers varied but they were mostly extensive, with a minimum size of 5 × 6.5 cm. Despite relatively massive destruction in the calcaneus in patients with Wagner grade III pedal ulcers, the extent of the bony debridement simplified the wound closure in these patients. The frequency of isolated pathogens was 34.7%, 22.3%, 26%, 13%, and 4% for *Staphylococcus aureus*, polymicrobial, *Ps*. *aeruginosa*, *Klebsiella pneumonia* *e*, and *Escherichia coli*, respectively.

At the final follow-up examination, complete clinical cure was seen to have been achieved in 18 patients (78%) with a painless and functional foot. All of these patients were able to ambulate without the need for a walking aid. However, at a mean of 8.4 weeks (range 7–9 weeks) after the procedure, three patients had partial recovery and still had to use a walker for ambulation, while two patients experienced proximal and distal advancement of the ongoing necrosis despite serial debridement. Although control MRI images and infection markers revealed cure of the osteomyelitis, the foot ulcers were classified as Wagner grade II in patients with partial recovery. Subsequent sural fasciocutaneous flaps were applied to these patients. After removal of the Ilizarov external fixator and further surgical debridement, the sural flap was rotated and secured to the defective area by the plastic and reconstructive surgery team in the same session. A split-thickness skin graft was used to cover the donor site in these patients. The external fixator did not complicate the flap coverage in any of these patients. Below-the-knee transtibial amputation was performed on the remaining two patients.

Pin (wire) site infection was slightly high; of 23 patients treated with this technique, pin site infection was seen in 16 patients (69.5%) on at least one wire; 14 (87%) of these were minor and two (13%) were major. While the minor infections responded to meticulous pin site care (*n* = 8, 57%) or oral antibiotics (*n* = 6, 43%), the major infections were treated by removal of the offending wires.

## Discussion

Severe diabetic foot infection is a major cause of morbidity and hospitalization. Some authors have stated that treatment of osteomyelitis in diabetic patients is time consuming because of vascular insufficiency, peripheral neuropathy, and poor soft-tissue coverage. Secondary infection from diabetic foot ulcers is a major reason for amputation. The most common pathogens that are isolated from the wounds are reported to be *S. aureus* (38.4%), *Ps. aeruginosa* (17.5%), and *Proteus* species (14%), and the most effective antibiotics for Gram-positive and Gram-negative bacteria have been reported to be vancomycin and imipenem, respectively [13]. The results of this study confirmed the above-mentioned incidence of pathogens. Treatment options for diabetic hindfoot ulcers have less than full cure-rate efficacy and have great potential for chronic osteomyelitis and ultimately amputation [14–17]. Local wound care, hospitalization for intravenous antibiotic therapy, and serial surgical debridements of the necrotic tissues have been advocated in the literature to prevent amputation. Currently, the most widely accepted treatment of diabetic calcaneal ulcers is a combination of serial debridements and partial calcanectomy [9,10,18–21]. Geertzen et al. and Perez et al. reported evidence supporting the role of calcanectomy in preventing amputations, which can be both a limb- and life-saving procedure [9,10]. However, in the literature there is a lack of studies with large numbers of cases concerning wound care and Ilizarov external fixator application, which is intended to simplify the wound closure in such patients. With the available number of cases, the current study can be considered to fill this gap and could guide future studies related to the prevention of amputation in diabetic calcaneal ulcers.

Preoperative assessment and planning are critical for the patient’s overall successful outcome. The surgeon must assess the extent of both the ulcer and osteomyelitis to make a surgical plan for debridement and osteomyelitis resection level. The diagnosis of osteomyelitis is often difficult to establish with plain radiographs [8]. A PTB test has been accepted as the most reliable technique to diagnose the presence of osteomyelitis in diabetic foot ulcers, although this test is unable to show the extent of the osteomyelitis. In addition, the extent of osteomyelitis may not be related to the area of the ulcer which has been probed [3]. MRI is more sensitive than plain radiographs and bone scans in detecting abscesses [22]. Yuh et al. reported that MRI had higher sensitivity and specificity than radionuclide studies in detecting osteomyelitis and in detecting osteomyelitis and the extent of involvement. Therefore, MRI is the modality of choice for the evaluation of pedal osteomyelitis and soft-tissue infection, with reported sensitivity of 90% and specificity of 83% [22]. Early detection of osteomyelitis and abscess formation in the diabetic foot is the first step towards successful treatment [23]. In the current series, MRI was used as a mapping tool to determine the extent of the ulcer and calcaneal osteomyelitis. This can be considered to be the main strength of this technique, by which a thorough debridement and calcaneal resection can facilitate the entire procedure. The size of the radical debridement can be planned up to the level of the endpoint of hypointensity on T1-weighted images and hyperintensity on fluid-sensitive images and the addition of 0.5 cm of bone. In a study by Braun et al., it was suggested that there is no significant benefit of surgical debridement over standard treatment, and this can be attributed to both a lack of preoperative MRI mapping of the ulcer and external fixation of the foot in a plantarflexion position. The relatively high satisfactory results of the current study seem to be related to both methods [24].

It is important to obtain definitive closure in a reasonable amount of time. After surgical debridement, the application of an Ilizarov external fixator allows the surgeon to approximate the wound ends easily and to monitor the wound healing during postoperative care. Prokuski and Saltzman reported the use of a monolateral external fixator to treat simultaneous bilateral ankle Charcot arthropathy in a young diabetic woman with severe osteopenia [25]. There are few reports about radical debridement, decreasing the soft-tissue defect, and stabilization with an external fixator. Diabetes mellitus has been considered as a relative contraindication for application of the Ilizarov method because of the high incidence of pin tract infection. However, the Ilizarov method is less invasive than other methods and can be used in septic conditions with excellent results [26–28]. For this reason, the Ilizarov external fixator was selected in this study and applied to the diabetic patients. The external fixator allowed wounds to be monitored and facilitated daily wound dressing changes. This technique also eliminated the use of a postoperative cast and the associated complications. Another advantage of the external fixator can be considered to be the relief of direct pressure over the heel, thereby facilitating wound healing.

The final outcome of this procedure in most patients is a healed but partially amputated heel. Partial foot amputations have many advantages over below-the-knee amputations. Often, a patient may walk without a special device and may require only a simple orthosis in the shoe. A patient with a partial foot amputation expends less energy than one with a below- or above-the-knee amputation. However, it is always preferable to retain as much of the foot as possible, as in this described technique [19,23].

## Study limitations

We acknowledge the limitations of this study. First, it describes a relatively expensive procedure with the use of both MRI and Ilizarov fixation in a group of diabetic patients with pedal ulcer. However, when considering amputation and the long-term consequences, such as dependency on orthosis usage and the consequent result of an index amputation for later amputations and higher mortality rates, this combination seems to be a rational procedure to manage soft-tissue defects and osteomyelitis. Another key weakness of this study is the small sample size, although the efficacy of the procedure was demonstrated by the high patient satisfaction rate. From this experience of a relatively limited number of patients, we evaluated the results of this surgical protocol, consisting of preoperative MRI, thorough surgical debridement, partial calcanectomy, and Ilizarov external fixation in plantarflexion of the ankle. Although relatively expensive, a salvage procedure instead of amputation was provided, with a satisfaction rate of 91%, when amputation was accepted as an undesirable final outcome. Finally, the chronic nature of diabetes mellitus leads to poor nutrition and ultimately lower levels of serum albumin in these patients. Hence, as the study was intended to report the effectiveness of the proposed technique, a dedicated study evaluating predictors of treatment failure in diabetic foot ulcers with calcaneal osteomyelitis may add more information to the literature.

## Conclusion

The current research presents a novel approach to the management of severe diabetic hindfoot ulcers and calcaneal osteomyelitis. MRI-guided resection up to where the hypointensity on T1-weighted images ends with the addition of up to 0.5 cm of spongy and fibrous bone enables radical debridement of the infection. Following thorough surgical debridement, Ilizarov external fixation facilitates wound closure and allows for serial surgical debridements where primary wound closure is not possible. This technique is simple and straightforward for surgeons compared to other salvage techniques.
